# Screening Tools Designed to Assess and Evaluate Oropharyngeal Dysphagia in Adult Patients: A Scoping Review

**DOI:** 10.3390/nursrep12020025

**Published:** 2022-04-02

**Authors:** Rafael A. Bernardes, Arménio Cruz, Hugo Neves, Vítor Parola, Nuno Catela

**Affiliations:** 1 The Health Sciences Research Unit: Nursing (UICISA:E), Nursing School of Coimbra (ESEnfC), Avenida Bissaya Barreto, 3004-011 Coimbra, Portugal; acruz@esenfc.pt (A.C.); hugoneves@esenfc.pt (H.N.); vitorparola@esenfc.pt (V.P.); 2 Portugal Centre for Evidence-Based Practice: A JBI Centre of Excellence, 3004-011 Coimbra, Portugal; 3 Polytechnic of Leiria—School of Health Sciences, Morro do Lena—Alto do Vieiro, 2411-901 Leiria, Portugal; nuno.correia@ipleiria.pt

**Keywords:** deglutition, deglutition disorders, review, outcome and process assessment

## Abstract

Oropharyngeal Dysphagia (OD) significantly decreases a patient’s quality of life and poses a high economic burden to institutions. In this sense, evaluation and assessment are important interventions for health professionals, although current tools and instruments are multiple and are dispersed in the literature. The aim of this review was to map existing screening tools to assess and evaluate OD in adult patients, identify their relevant clinical parameters and respective contexts of use and provide a systematic approach and summary to better inform practice. A scoping review was developed guided by the JBI methodology and using PRISMA-ScR to report results published between 2014 and 2021, in English, Spanish and Portuguese. Databases included Medline, Academic Search Complete, CINAHL Complete, Scielo, Google Scholar, ScienceDirect, OpenGrey and B-On. Mendeley was used to store and screen data. A total of 33 studies were included in the study, of which 19 tools were identified, some being intervention-based tools and others an algorithm for decision. The most common context used was in the general population and older adults. Regarding clinical parameters, the most common were food consistency, presence of the cough reflex, swallowing effort, voice changes and weight. As oropharyngeal dysphagia concerns important risks for the patient, a rigorous assessment must be performed. In this sense, the review identified specific disease-related tools and more general instruments, and it is an important contribution to more efficient dysphagia screening and prevention.

## 1. Background

The swallowing process is highly complex, involving six cranial nerves and several muscle groups, being traditionally divided into three phases: oral, pharyngeal and esophageal [1]. Significant changes in the swallowing process can often occur, resulting from several underlying pathological processes. In this sense, dysphagia can be defined as an abnormal delay or misdirection in the transit of a liquid or solid bolus during the oropharyngeal or esophageal swallowing stages [2]. The most common causes are stroke, head and neck neoplasms and progressive neurological diseases, such as dementia [3].

This review focused on oropharyngeal dysphagia (OD), since its prevalence is very high, affecting 7% to 13% of those aged 65 or older [4]. OD is present in over 30% of patients with stroke, 52–82% of patients with neurodegenerative diseases, 30% of patients with head and neck diseases and 60% of elderly in hospitals [5].

Furthermore, OD is particularly common among frail older people, being described by the European Society for Swallowing Disorders (ESSD) as a geriatric syndrome [6], usually presenting multiple age-related changes, like loss of muscle mass and sarcopenia [7]. Additionally, critical complications may arise from dysphagia, namely aspiration pneumonia [7,8,9], one of the most prominent death causes in older adults, associated with mortality rates up to 50% [10]. Recently, a case report focused on OD and related pneumonia aspiration, following infection by SARS-CoV-19, and it concluded that the patient developed impaired pharyngolaryngeal sensation, mesipharyngeal contractile dysfunction and silent aspiration in the recovery phase [11], thus eliciting the need to early detect and prevent OD events. More recently, Seo and colleagues [12] concluded that there is a high prevalence of OD in patients hospitalized for aspiration pneumonia, highlighting the need to diagnose and evaluate OD, even regardless of neurologic disorders. Thus, adequate screening of dysphagia and further management should be mandatory [13].

Additionally, after the decrease in patients’ quality of life and increase in mortality rates, there is a high burden on healthcare services. A recent systematic review [14] concluded that economic costs increase during hospitalization and long-term follow-up in patients who developed post-stroke OD.

The preliminary evaluation of the aspects inherent to the swallowing process aims to describe the general aspects of the breathing, motor and sensory alterations. Among the main clinical measures considered, there are clinical diagnosis data, orientation status, alertness/awareness, ability to obey simple orders, changes in vital signs, presence of feeding tubes, O_2_ saturation, breathing pattern, dentition characteristics, presence of changes in speech, voice changes, cough quality, laryngeal elevation, presence of saliva and inability to swallow it, among other signs and symptoms [15].

Dysphagia assessments may involve the use of the Fibreoptic Endoscopic Evaluation of Swallowing (FEES), Videofluoroscopic Swallowing Study (VFSS) or diagnostic ultrasound. Although being considered the ‘gold standard’, these procedures are invasive and expensive, also involving radiation exposition and risk of aspiration. Moreover, they can be less clinically relevant as they cannot be carried out frequently for possible reevaluations due to specialized equipment and qualified personnel requirements, increasing the related costs and morbidity [16,17].

In the last years, the ESSD has published specific Position Statements related to OD in adult patients [18] and also in stroke patients [19], with established guidelines for OD management, which have been implemented in many healthcare professionals’ routine as a safety measure when feeding patients with significant risk factors or changes in deglutition.

When referring to non-invasive assessments and interventions, Costa [18] mentions that they are reliable, fast, associated with less discomfort and showing promising results. Thus, the importance of implementing work tools that allow healthcare professionals to carry out an adequate and accurate swallowing assessment and early implementation of preventive interventions must be noted [9].

It should be noted that although swallowing disorders are frequently detected in clinical practice, multiple evaluation and assessment tools are found in the literature [20], leading to a dispersion of this knowledge.

In this sense, this review aims to map existing screening tools to assess and evaluate OD in adult patients, identify their relevant clinical parameters and respective contexts of use and provide a systematic approach and summary to better inform practice. A research question was developed considering the PCC mnemonic proposed by JBI [21], standing for Population, Concept and Context. The review intended to answer the following question: ‘Which are the currently used screening tools to evaluate and assess oropharyngeal dysphagia and their respective clinical parameters and context applied?’.

## 2. Materials and Methods

### 2.1. Study

This scoping review followed the Preferred Reporting Items for Systematic reviews and Meta-Analysis extension for Scoping Reviews (PRISMA-ScR) [2] and was guided by the methodology proposed by JBI to adequately conduct scoping reviews [21,22,23,24].

An initial search of MEDLINE (PubMed), the Cochrane Database of Systematic Reviews, the JBI Evidence Synthesis, PROSPERO and Open Science Framework (OSF) revealed no conducted, current or underway scoping or systematic reviews that address this topic.

### 2.2. Literature Search

A preliminary search related to the topic was developed to identify relevant keywords. Databases searched included Medline (PubMed), Academic Search Complete (EBSCO), CINAHL Complete (EBSCO), Scielo, Google Scholar, ScienceDirect, OpenGrey and B-On. The search strategy used for MEDLINE (PubMed) is presented in Table 1. The queries were further adapted to each specific thesaurus. The last database searched was OpenGrey on 1 December 2021.

Since there is a high amount of primary information, we considered that the most adequate methodology was to search for records published in the last seven years (between 2014 and 2021). Furthermore, this would have led to a more immediate access to the most recent evidence, which is more likely to portray the world’s current reality. Additionally, this timeframe is consistent with the view that scoping reviews consists of a crucial link between theory and evidence-based decision. This perspective contributes to develop a reliable and valid review, thus being helpful [25,26,27,28,29]. Moreover, a systematic review by Etges et al. (2014) [30] has described screening tools for dysphagia, which helped the definition of an adequate timeframe. Following that year, new tools might have been developed and others have been abandoned due to newer findings and challenges posed to healthcare professionals. In this sense, our review constitutes an important update about OD screening and assessment in the last years. Documents published in English, Spanish and Portuguese were included.

The search strategy was designed to the specificities of each information source. Furthermore, retrieved publications’ reference lists were screened to potentially include studies that met the inclusion criteria.

### 2.3. Inclusion and Exclusion Criteria

The present review considered the following inclusion criteria: (i) tools specifically designed to assess and evaluate OD in adult patients.

The following exclusion criteria were considered: (i) tools that measure patients’ quality of life; (iii) instruments to measure patients’ perspectives about dysphagia; (iv) assessment and evaluation of OD using invasive methods (e.g., FEES, VFSS) and (v) pediatric dysphagia assessment tools (pediatric versions).

The PRISMA flow chart [27] explained the reviewing process and respective phases.

### 2.4. PCC Mnemonic: Population, Concept, Context

Regarding population, the review considered studies that included screening tools to assess and evaluate OD in adult patients, regardless of the condition associated.

As for concept, studies addressing screening tools specifically designed to assess and evaluate OD in adult patients and respective clinical evaluation parameters were included.

Regarding the context, this review considered studies targeting any adult healthcare setting.

### 2.5. Study Selection and Screening Process

All the records found through database searching were retrieved and stored in Mendeley^®^ V1.19.8 (Mendeley Ltd., Elsevier, The Netherlands), and duplicates were removed. After a pilot test, retrieved titles were screened by two reviewers independently, considering the inclusion and exclusion criteria. Full reports of the titles and abstracts that met the criteria were obtained for a second screening against inclusion and exclusion criteria. Disagreements between reviewers were resolved through discussion or with a third reviewers.

## 3. Results

**Figure 1:**
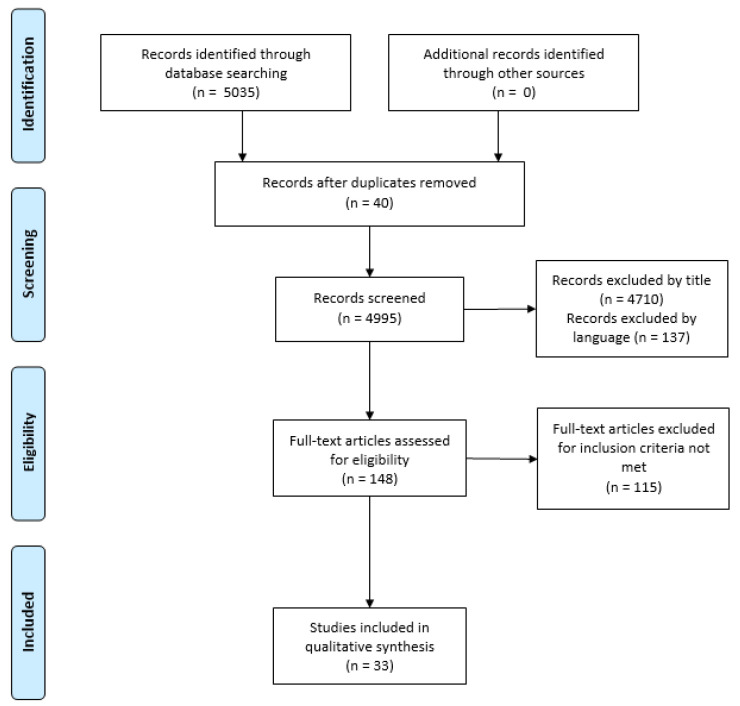
PRISMA Flowchart.

A total of 5535 studies were retrieved from the main databases considered for the review. After the identification and screening phases of the review process (Figure 1), a total of 33 studies were included, having met the inclusion criteria previously defined.

### 3.1. Study Characteristics

The characteristics of the included studies are summarized in Table 2. The 33 studies were published between 2014 and 2020 and were from Iran (*n* = 1), U.S.A (*n* = 3), Germany (*n* = 2), Denmark (*n* = 3), Canada (*n* = 1), Japan (*n* = 5), Italy (*n* = 3), Sweden (*n* = 1), Israel (*n* = 1), Turkey (*n* = 2), Belgium (*n* = 2), Colombia (*n* = 1), Israel (*n* = 1), Portugal (*n* = 1), Korea (*n* = 2), Sweden (*n* = 1) and Spain (*n* = 1), and one was a multicenter study.

The review allowed for the identification of 19 OD assessment and evaluation tools: Swallowing Disturbance Questionnaire in Parkinson’s Disease Patients (SDQPD), Mann Assessment of Swallowing Ability (MASA), McGill Ingestive Skills Asessment (MISA), Toronto Bedside Swallowing Screening Test (TOR-BSST), Facial-Oral Tract Therapy Swallowing Assessment of Saliva (FOTT-SAS), Eating Assessment Tool (EAT-10), Kuchi-Kara Taberu Index, Saku-Saku Test, Gugging Swallowing Screen (GUSS), Royal Birsbane and Women’s Hospital (I-RBWH) Dysphagia Screening Tool, Postextubation Dysphagia Screening (PEDS) Tool, Test of Masticating and Swallowing Solids (TOMASS), Repetitive Saliva Swallowing Test (RSST), Questionnaire for the Assessment of Dysphagia in Multiple Sclerosis (QAD-MS), Sydney Swallow Questionnaire (SSQ), Assessment of Swallowing Ability for Pneumonia (ASAP), Oral and Maxillofacial Frailty Index (OMFI), Oropharyngeal Dysphagia Screening Test for Patients and Professionals (ODS-PP) and Mealtime Assessment Tool (MAT).

### 3.2. Tool’s Structure

The identified tools present a heterogeneous structure, with different types and number of items, with some being a clear intervention-aid tool for professionals, like the PEDS [28] or ASAP [30], which offer algorithms for decision, and others being a self-reported questionnaire, like the SSQ [31]. Interestingly, the MAT [32] has a specific application for caregivers, which seems a clear innovation among the topic in study.

The most frequently mentioned tool was the EAT-10, which in the last seven years was validated in five different languages: Swedish [33], Hebrew [34], Turkish [35], Spanish [36] and Japanese [37].

Table 3 presents each tool categorized according to the ESSD white paper [6].

### 3.3. Contexts Where Tools Are Used

Regarding the contexts where dysphagia tools are usually applied, Table 4 resumes the most common context where the identified tools were validated. The most common application is in the general population and older adults, followed by head and neck cancer and stroke patients. There are tools with a specific focus, like the SDQPD for Parkinson’s Disease, the PEDS for extubated patients, the QAD-MS for Multiple Sclerosis or the SSQ for Muscular Dystrophies.

### 3.4. Clinical Parameters

Regarding clinical parameters, those that are more frequently considered, either in response to liquid or solid food, seem to be the cough reflex, drooling while eating or drinking, swallowing effort, swallowing frequency, voice changes, breathing difficulties, food sticking in the throat and weight loss/gain. Subjective parameters are related to pleasure during eating, going out for meals and desire to eat.

## 4. Discussion

The present review addressed the following research question: ‘Which are the currently used screening tools to evaluate and assess oropharyngeal dysphagia and their respective clinical parameters and context applied?’.

Currently, OD is being underreported in clinical settings [28], hindering care planning. In this sense, early detection is important for adequate prevention, patient rehabilitation and health promotion. Moreover, the early detection of dysphagia helps prevent complications during hospitalization and the possible increase in the length of stay in hospitals [30]. In fact, one of the most common complications is aspiration pneumonia, leading to increased morbidity and mortality rates among institutionalized older adults [9].

This review highlighted the need to assess respiratory, motor and sensory changes before the application of any specific assessment instrument. In addition, attention should be given to other aspects, such as positioning, diet adequacy and head flexion.

According to the mapped literature, assessment instruments should be used only after the preliminary assessment, preferably a non-invasive intervention, as recently demonstrated by a prospective exploratory study [31].

EAT-10, a self-report instrument, was the most commonly referred to tool in the literature, and according to some studies [32,33], it is also able to predict aspiration risk, which might lead to conclude that some of the most important clinical parameters for dysphagia screening are those portrayed by EAT-10: weight, going out for meals, solid-liquid, pills, pain while swallowing, pleasure, food in throat, cough and stress during swallowing. The increasing number of studies related to EAT-10, a community-based instrument, might be linked with new healthcare trends, where the patient is treated in his community or even in his home. This has been highlighted by several institutions, advocating a major shift of a paradigm from acute settings (e.g., hospitals) to long-term management in the community [34].

Together with dysphagia screening performed by nurses, for example, the use of these type of instruments and the prediction of some risks and complications might be associated with dysphagia etiology [35]. This entails the importance of communication between different disciplines, as this is a determinant of quality and safety in the provision of care to dysphagic patients [36]. As awareness about dysphagia signs and symptoms has increased over the years, the need to perform an accurate and efficient screening is crucial. As such, together with a patient-centered approach, interprofessional management is needed [36], namely gathering inputs from nutritionists, speech therapists, nurses, medical doctors, families, physiotherapists and other relevant professionals involved in the care process. This is also highlighted by the ESSD, which specifically states that dysphagia should be managed by multidisciplinary teams [19].

The tools found usually perform a distinction between liquid, semiliquid and solid foods and also the presence of food in the oral cavity. Although this is a common parameter found in almost all tools, there seems to be very little evidence about consistency and the influence on swallowing function and physiology. Other properties should be considered, like cohesiveness, hardness and slipperiness [38]. A study by Sukkar and colleagues [39] investigated the possible influence of these types of new properties, like cohesiveness, and the influence of saliva in the development of a bolus, posing that this is critical for OD.

Beyond these parameters, relevance has been given to the difficulty to chew, presence of cough or voice changes, as in the SDQPD and TOR-BSST scales, or positioning, as in the MISA-DK.

Some tools, such as the MASA-C, FOTTS, modified MASA, PEDS Tool and ASAP, focused on alertness and consciousness, which were already highlighted as predictors of severe dysphagia. Particularly, the oral phase is severely altered in patients with disorders of consciousness, hence the importance of including this type of variable in dysphagia screening tools [36]. Recently, a study [40] has given importance to the initiation of swallowing, which is related to disorders of consciousness and increased risk for dysphagia complications. The time taken to swallow was only mentioned in three of the tools (the SDQPD, MASA-C and TOMASS), which might elicit the need to improve the existing instruments.

Interestingly, the EAT-10, Kuchi-Kara and ODS-PP tools mention subjective measures about dysphagia, namely the desire to eat or going out for meals, which seems to be an important component of clinical evaluation, as dysphagia significantly influences the patient’s quality of life [41]. In fact, recent studies have highlighted how dysphagia greatly impacts quality of life [42,43], namely highlighting that rehabilitation and treatment should focus on the pharyngeal phase of dysphagia. Furthermore, patients’ perspectives play a crucial role in treatment, namely through adequate communication throughout the process [44]. Further research and studies should address how instruments and tools can include these subjective measures as essential parameters for an efficient diagnostic and preventive measure.

Finally, the developed instruments are more commonly applied in the general population, with a clear focus on older patients, but the existence of specific tools are an advantage for healthcare services. Furthermore, although many different contexts were explored, there seems to be a lack of well-validated self-report questionnaires for progressive neurological disorders [45,46]. To some extent, applying self-reported tools, like the ODS-PP, to the population with a certain degree of cognitive deficits might elicit some important limitations on the efficacy of diagnosis and prevention. Although this is, it is a motivation to increase caregivers’ health literacy, to which the MAT tool offers a great contribution, since it is specifically for caregivers. Furthermore, it strengthens the importance of the family and partner as having an active role during all disease management processes.

Taking into account the review developed by Etges and colleagues [29], we can highlight that some tools have been continuously used throughout the years, like the EAT-10 or MASA, and others have been less mentioned in the literature.

Given the above, all health professionals must have specific training, knowledge and experience in using different assessment methods and intervention techniques in the context of swallowing disorders. Their handling must be carried out in a multidisciplinary team and in a complementary way.

Regarding study limitations, although the time limit span was chosen to obtain the most recent evidence, this is still a limitation of the present scoping. Additionally, the search strategy could be further refined to allow the possible identification of other instruments. Furthermore, it would be interesting to increase the search in more databases.

## 5. Conclusions

The study of the assessment and evaluation of OD is particularly important, as it presents as a heavy burden for institutions, professionals, patients and respective families.

Complications are usually a cause of wrong or inefficient screening of relevant clinical parameters that are a risk for the development of severe dysphagia and, more importantly, aspiration.

The discussion of which are the available tools for this purpose turns out to be a necessity when the literature has numerous instruments, which are scattered and might be difficult to find, to explore the clinical parameters evaluated and contexts where they are or should be used.

In this sense, this review allowed for the identification of 19 different tools to screen and evaluate OD, thus contributing to inform practice, giving professionals and institutions relevant and robust findings for a structured and informed decision about OD screening.

## Figures and Tables

**Table 1:** Search strategy for MEDLINE (PubMed) conducted on 3 March 2021.

Search	Query	Records Retrieved
#1	screening [Title/Abstract] OR assessment [Title/Abstract] OR test [Title/Abstract] OR diagnosis [Title/Abstract] OR tools [Title/Abstract]	244,638
#2	dysphagia [Title/Abstract] OR swallowing disorders [Title/Abstract] OR deglutition disorders [Title/Abstract] OR swallowing [Title/Abstract] OR swallow [Title/Abstract]	3034
#3	adults [Title/Abstract] OR adult [Title/Abstract] OR aged [Title/Abstract] OR elderly [Title/Abstract]	183,689
#4	#1 OR #3	183,689
#5	#1 OR #2	6741
#6	#2 OR #3	5321
#7	#4 AND #5 AND #6	1273

**Table 2:** Summary of the included studies (*n* = 33).

Author (s), Year and Country	Tool’s Name	Clinical Parameters	Structure	Contexts Validated
Rajaei et al., 2014, Iran	Swallowing Disturbance Questionnaire in Parkinson’s Disease Patients (SDQPD)	Chewing (solid food); food residues; food or liquid coming out of nose or mouth while eating; dribbling; saliva drooling or difficult swallowing; swallowing several times; difficult swallowing (solid food); cough; voice changes; difficult breathing; history of respiratory infection.	Likert-type questionnaire with 15 questions, ranging from 0 (‘never’) to 3 (‘very frequently’).	Parkinson’s patients
Carnaby and Crary, 2014, USA	Cancer-Specific Swallowing Assessment Tool (MASA-C)	General patient examination (alertness, cooperation, auditory comprehension, aphasia, apraxia, dysarthria); oral preparation phase (saliva, lip seal, tongue movement, tongue strength, tongue coordination, oral preparation, respiration, respiratory rate for swallowing); oral phase (gag reflex, palatal movement, bolus clearance, oral transit time); pharyngeal phase (cough reflex, voluntary cough, voice tracheostomy, pharyngeal phase, pharyngeal response). Neck palpation, mouth opening, taste, smell, current diet, oral mucous membrane, weight loss.	Score with 24 items measured using a 5-point to 10-point Likert-type scale. The total score of the MASA is 200 points.	Patients undergoing radiotherapy for head/neck cancer.
Giselle et al., 2014, Germany	Mann Assessment of Swallowing Ability—Cancer (MASA—C)			
Hansen, 2014, Denmark	McGill Ingestive Skills Assessment (MISA-DK)	Positioning; Self-feeding skills; Liquid ingestion; Solid ingestion	Composed of 36 ingestive skill items distributed into four subscales. Scored on a three-point ordinal scale (1 = ‘absent ingestive’; 3 = ‘adequate ingestive skill performance’).	Measures eating and drinking in elderly dysphagic patients.
Martin et al., 2014, Canada	Toronto Bedside Swallowing Screening Test (TOR-BSST)	Voice quality (before and after); tongue movement; water swallows.	Includes 10 teaspoons of water (5 mL), swallows and adds a cup sip of those who passed all previous 10 teaspoons.	Stroke patients.
Mortensen et al., 2015, Denmark	Facial-Oral Tract Therapy Swallowing Assessment of Saliva	Conscious and/or response to verbal address; able to sit upright with some head control; oral transport of saliva; spontaneous or facilitated swallowing of saliva; coughing following swallowing of saliva; gurgling breath sound following swallowing of saliva; difficulties breathing following swallowing of saliva.	Seven-item scale with a combination of swallowing and non-swallowing items.	Adult patients with acquired brain injury.
Maeda et al., 2016, Japan	Kuchi-Kara Taberu Index	Desire to eat; overall condition; respiratory condition; oral condition; cognitive function while eating; oral preparatory and propulsive phases; dysphagia severity; position and endurance while eating; daily life; food intake level; food modification; nutrition.	A total of 13 items regarding physical, nutritional and medical conditions. Each item is rated from 1 (worst) to 5 (best) points.	English version for older adults (aged 65 and older).
Mozzanica et al., 2017, Italy	Royal Brisbane and Women’s Hospital (I-RBWH) Dysphagia Screening Tool	Conditions commonly linked to dysphagia (e.g., COPD, stroke, neurological involvement, HNC); signs of dysphagia or aspiration risk (e.g., altered level of alertness, slurred speech, weak/absent volitional cough, weak voice, difficulty swallowing).	Consists of three steps: (1) two-phase question screen; (2) water swallow test, as appropriate; (3) swallowing management plan.	Dysphagia screening of patients in subacute settings and generic acute hospital use
Möller et al., 2016, Sweden	Eating Assessment Tool (S-EAT-10)	Weight loss; going out for meals; swallowing liquids; swallowing solids; swallowing pills; swallowing is painful; pleasure of eating; food sticking in the throat; coughing when eating; swallowing is stressful.	Likert-type 10 statement questionnaire, scored 0 to 4, where 0 is ‘no difficulty’ and 4 ‘severe difficulty’.	Swedish population; quantifies swallowing problems and treatment efficacy in general populations.
Abu-Ghanem et al., 2016, Israel	Hebrew Version of the Eating Assessment Tool-10 (H-EAT-10)			Hebrew-speaking population. Measures dysphagia symptoms severity and effects on quality of life.
Arrese et al., 2016, USA	Eating Assessment Tool-10 (EAT-10)			Individuals with head and neck cancer (HNC)
Demir et al., 2016, Turkey	Turkish Eating Assessment Tool (T-EAT-10)			Translated to Turkish, used in neurogenic dysphagia patients.
Giraldo-Cadavid et al., 2016, Colombia	Eating Assessment Tool-10 (EAT-10)			Spanish version
Sara et al., 2017, Israel	Eating Assessment Tool-10 (H-EAT-10) Hebrew Version			Hebrew-speaking population; measures dysphagia symptom severity and effects on quality of life.
Nishida et al., 2019, Japan	Eating Assessment Tool-10 (EAT-10)			Older individuals use the Japanese version.
Hansen and Kjaersgaard, 2020, Denmark	Eating Assessment Tool-10 (EAT-10)			Clinical populations and non-clinical populations of community-dwelling elders (multiple studies)
Warnecke et al., 2017, Germany	Gugging Swallowing Screen (GUSS)	Swallowing screening tests: uses multiple consistencies, starting with pudding to reduce the risk of aspiration to a minimum during the screening procedure and to allow a graded stepwise assessment; screen aspiration risk; offers dietary recommendations.	Composed of two parts: a non-swallow clinical screening test followed by a direct bolus-swallowing screening test.	Patients with stroke; NIHSS score 0–4, 5–9, 10–14 and >15.
Ferreira et al., 2018, Portugal	Gugging Swallowing Screen (GUSS)			Patients with cardiac, respiratory, neurological and cancer diseases who were admitted to a medicine ward.
Ohira et al., 2017, Japan	Mann Assessment of Swallowing Ability (MASA)	General patient examination; oral preparation; oral phase and pharyngeal phase.	It is comprised of 24 clinical parameters.	Dependent older adults with comorbidities.
Ji et al., 2019, Korea	Modified Mann Assessment of Swallowing Ability	Alertness; cooperation; respiration; expressive dysphasia; auditory comprehension; dysarthria; saliva; tongue movement; tongue strength; gag; voluntary cough and palate movements.	A total of 12 clinical items (weighted scoring depends on the item).	Patients with mild to moderate dementia and dysphagia
Tagashira et al., 2017, Japan	Saku-Saku Test	Chewing; Swallowing	Patient is asked to eat a 2 g rice cracker to evaluate the quality of mandibular rotation during mastication while sitting	Patients who could consume thickened liquids in a sitting position without aspiration, who did not have a history of choking or had not undergone surgery for oral cancer, and who were conscious.
Johnson et al., 2018, U.S.A.	Postextubation Dysphagia Screening (PEDS) Tool	Level of alertness; respiratory status (CPAP or BiPAP support; saturation levels; respiratory rate); presence of feeding tubes (oral-gastric, nasal gastric); history of dysphagia; adverse lung sounds; voice changes; history of head/neck trauma; swallowing difficulty; cough; weight loss and dehydration; history of head/neck cancer; history of stroke	Five sections with a decision algorithm-type structure	Determines an extubated patient’s ability to swallow after prolonged endotracheal intubation
Huckabee et al., 2018, Multicentered Study	Test of Masticating and Swallowing Solids (TOMASS)	Number of bites, number of masticatory cycles, total time taken and number of swallows	Quantitative score (in seconds) per item	Clinical assessment of solid bolus ingestion; provides a measure of functional change in swallowing.
Persson et al., 2018, Sweden	Repetitive Saliva Swallowing Test	Larynx movement	Asked to swallow their own saliva as many times as possible in 30 s.	Young adult patients to older participants and older strokes patients
Tenekeci et al., 2018, Turkey	Turkish version of the questionnaire for the assessment of dysphagia in multiple sclerosis (DYMUS)	Self-perceived OD (Food sticking; Needs several swallowing actions to swallow solids; difficulty swallowing solids; globus sensation; cuts food into small pieces to swallow; coughing after ingestion of solids; weight loss; difficulty swallowing liquids; coughing after ingestion of liquids)	Two Subdimensions, i.e., dysphagia for solids (items 1, 3, 4, 5, 7, 8 and 10) and dysphagia for liquids (items 2, 6 and 9). All the items of the scale are coded as “No = 0” and “Yes = 1,” and the total scale score varies between 0 and 10. Dysphagia is diagnosed if the score is ≥1.	Multiple sclerosis patients treated as inpatients at a neurology clinic of a training hospital or who had presented at the outpatient department (Turkish validation)
Quirtós et al., 2020, Spain	Oropharyngeal Dysphagia Screening Test for Patient and Professionals (ODS-PP)	Shortness of breath or difficulty breathing while eating; cough during and/or after eating; avoiding solid intake; avoiding liquid intake; voice changes after eating; need to clear throat; phlegm or mucus; lost weight in last 6 months; saliva retention; food debris after eating; food/liquid dribbling; problems to be understood by others; enjoyable eating; uncomfortable eating out; difficult swallowing; trouble swallowing pills; time to eat.	A total of 18 questions which are Likert-type, rated 1 to 4, where 1 is never, and 4 is very often.	Spanish-speaking population; people with cognitive disorders.
Omori et al., 2019, Japan	Assessment of Swallowing Ability for Pneumonia (ASAP)	Consciousness; Vocalization; Cough; tongue muscle; swallowing; thickened water; jelly; water.	Eight items with heterogeneous scores. Algorithm-type tool.	Elders with pneumonia.
Audag et al., 2019, Belgium	Sydney Swallow Questionnaire (SSQ)	Difficulty swallowing: thin/thick liquids; soft/hard foods; difficult swallowing saliva; difficult starting to swallow; food getting stuck in the throat; cough or choking when swallowing solid foods or liquids; duration to eat an average meal; food going up behind the nose or coming out of nose; number of swallows; cough or spit during the meal.	A 17-question, self-reported questionnaire using a visual scale for the detection and quantification of an oropharyngeal dysphagia inventory with a maximum total score of 1700.	Patients with impaired swallowing
Audag et al., 2019, Belgium	French Version of the Sydney Swallow Questionnaire (SSQ)			
Choi et al., 2019, Korea	Oral and Maxillofacial Frailty Index (OMFI)	Pain and/or bleeding in the tooth or gum; difficulties in chewing; necessity of water when eating dry food; jaw pain or difficulties in opening the mouth; difficulties in pain perception; difficulties in jaw or tongue movements; difficulties in speaking or pronunciation; difficulties swallowing.	A 10-item Likert-type questionnaire	Evaluation of oral and maxillofacial frailty in older adults
Gandolfo et al., 2019, Italy	Predictive Dysphagia Score (PreDyScore)	Personal medical history; associated diseases; stroke characteristics (type, site lesion, etiology); Bamford’s classification (7th and 30th days after admission); detection and evaluation of the degree of dysphagia (7th and 30th days after admission); evaluation of malnutrition (subjective global assessment); nutritional therapy (parenteral/enteral); type of products used for artificial feeding.	3-oz water swallow test performed on the first day and on the 7th and 30th days.	Predictive score for persistent dysphagia in stroke patients
Rossi et al., 2020, Italy	Mealtime Assessment Tool (MAT)	Demographic data (age, gender, primary pathology, weight and height); patient’s participation; posture during meal; presence of distractors; person’s autonomy; hydration; taste and appetite of patient; focus on the activity; cough; voice changes; presence of residues on lips, tongue and nostrils.	A total of 12 Likert-type questions	Italian study; specific for caregivers.

Note. SDQPD—Disturbance Questionnaire in Parkinson’s Parkinson’s Disease Patients; MASA—Mann Assessment of Swallowing Ability; EAT10—Eating Assessment Tool; MISA—McGill Ingestive Skills Assessment; TOR-BSST—Toronto Bedside Swallowing Screening Test; FOOT-SAS—Facial-Oral Tract Therapy Swallowing Assessment of Saliva; GUSS—Gugging Swallowing Screen; I-RBWH—Royal Brisbane and Women’s Women’s Hospital; PEDS—Postextubation Dysphagia Screening; TOMASS—Test of Masticating and Swallowing Solids; RSST—Repetitive Saliva Swallowing Test; SSQ—Sydney Swallow Questionnaire; QAD-MS—Questionnaire for the Assessment of Dysphagia in Multiple Sclerosis; ASAP—Assessment of Swallowing Ability for Pneumonia; OMFI—Oral and Maxillofacial Frailty Index; ODS-PP—Oropharyngeal Dysphagia Screening Test for Patients and Professionals; MAT—Mealtime Assessment Tool.

**Table 3:** Categorization of OD tools.

		Clinical Evaluation of Textures
Patient Reported Tools (Self-Administered)	SDQPD	
	EAT-10	x
	SSQ	x
	ODS-PP	
Observational Tools	MASA	
	MISA	x
	TOR-BSST	
	FOOT-SAS	
	Kuchi-Kara	
	Saku-Saku	
	GUSS	x
	I-RBWH	
	PEDS	
	TOMASS	
	RSST	
	QAD-MS	x
	ASAP	
	OMFI	
	ODS-PP	x
	MAT	

Note. MASA—Mann Assessment of Swallowing Ability; EAT10—Eating Assessment Tool; MISA—McGill Ingestive Skills Assessment; TOR-BSST—Toronto Bedside Swallowing Screening Test; FOOT-SAS—Facial-Oral Tract Therapy Swallowing Assessment of Saliva; GUSS—Gugging Swallowing Screen; I-RBWH—Royal Brisbane and Women’s Women’s Hospital; PEDS—Postextubation Dysphagia Screening; TOMASS—Test of Masticating and Swallowing Solids; RSST—Repetitive Saliva Swallowing Test; SSQ—Sydney Swallow Questionnaire; QAD-MS—Questionnaire for the Assessment of Dysphagia in Multiple Sclerosis; ASAP—Assessment of Swallowing Ability for Pneumonia; OMFI—Oral and Maxillofacial Frailty Index; ODS-PP—Oropharyngeal Dysphagia Screening Test for Patients and Professionals; MAT—Mealtime Assessment Tool.

**Table 4:** Contexts where OD screening instruments are used.

	General Population	Parkinson	HNC Patients	Older Adults	Stroke	Dependent Older Adults	Subacute Settings	Extubated Patients	Multiple Sclerosis	Unilateral Vocal Fold Paralysis	Muscular Dystrophias	Dementia/Cognitive Disorders	Elders with Pneumonia	Community
SDQPD		x												
MASA			x			x						x		
EAT-10	x		x	x						x				x
MISA				x										
TOR-BSST					x									
FOOT-SAS					x									
Kuchi-Kara				x										
Saku-Saku	x			x										
GUSS	x				x									
I-RBWH							x							
PEDS								x						
TOMASS	x			x										
RSST				x	x									
SSQ	x		x	x							x			
QAD-MS									x					
ASAP													x	
OMFI	x			x										
ODS-PP				x		x						x		
MAT	x													

Note. HNC—Head and Neck Cancer; PD—Parkinson’s Disease; MASA—Mann Assessment of Swallowing Ability; EAT10—Eating Assessment Tool; MISA—McGill Ingestive Skills Assessment; TOR-BSST—Toronto Bedside Swallowing Screening Test; FOOT-SAS—Facial-Oral Tract Therapy Swallowing Assessment of Saliva; GUSS—Gugging Swallowing Screen; I-RBWH—Royal Brisbane and Women’s Women’s Hospital; PEDS—Postextubation Dysphagia Screening; TOMASS—Test of Masticating and Swallowing Solids; RSST—Repetitive Saliva Swallowing Test; SSQ—Sydney Swallow Questionnaire; QAD-MS—Questionnaire for the Assessment of Dysphagia in Multiple Sclerosis; ASAP—Assessment of Swallowing Ability for Pneumonia; OMFI—Oral and Maxillofacial Frailty Index; ODS-PP—Oropharyngeal Dysphagia Screening Test for Patients and Professionals; MAT—Mealtime Assessment Tool.

## Data Availability

Data sharing is not applicable to this article.
